# The Cell Neural Adhesion Molecule Contactin-2 (TAG-1) Is Beneficial for Functional Recovery after Spinal Cord Injury in Adult Zebrafish

**DOI:** 10.1371/journal.pone.0052376

**Published:** 2012-12-21

**Authors:** Jin-Fei Lin, Hong-Chao Pan, Li-Ping Ma, Yan-Qin Shen, Melitta Schachner

**Affiliations:** 1 Center for Neuroscience, Shantou University Medical College, Shantou, People’s Republic of China; 2 Keck Center for Collaborative Neuroscience and Department of Cell Biology and Neuroscience, Rutgers University, Piscataway, New Jersey, United States of America; Beijing Institute of Radiation Medicine, China

## Abstract

The cell neural adhesion molecule contactin-2 plays a key role in axon extension and guidance, fasciculation, and myelination during development. We thus asked, whether contactin-2 is also important in nervous system regeneration after trauma. In this study, we used an adult zebrafish spinal cord transection model to test the functions of contactin-2 in spinal cord regeneration. The expression patterns of contactin-2 at different time points after spinal cord injury were studied at the mRNA level by qPCR and in situ hybridization, and contactin-2 protein levels and immunohistological localization were detected by Western blot and immunofluorescence analyses, respectively. Contactin-2 mRNA and protein levels were increased along the central canal at 6 days and 11 days after spinal cord injury, suggesting a requirement for contactin-2 in spinal cord regeneration. Co-localization of contactin-2 and islet-1 (a motoneuron marker) was observed in spinal cords before and after injury. To further explore the functions of contactin-2 in regeneration, an anti-sense morpholino was used to knock down the expression of contactin-2 protein by application at the time of injury. Motion analysis showed that inhibition of contactin-2 retarded the recovery of swimming functions when compared to standard control morpholino. Anterograde and retrograde tracing at 6 weeks after injury showed that knock down of contactin-2 inhibited axonal regrowth from NMLF neurons beyond lesion site. The combined observations indicate that contactin-2 contributes to locomotor recovery and successful regrowth of axons after spinal cord injury in adult zebrafish.

## Introduction

Contactin-2, also called transient axonal glycoprotein-1 (TAG-1) or axonin-1, belongs to the contactin, L1 and immunoglobulin superfamilies, with six immunoglobulin-like domains and four fibronectin type III homologous repeats [1]. Contactin-2 is attached to the membrane via glycan phosphatidyl inositol (GPI) anchoring and can be secreted into the extracellular matrix [2], [3]. Extensive studies have found contactin-2 to be spatially and temporally regulated in an array of neurons and glial cells during nervous system development, and to play a role in various morphogenetic functions. At formative stages of nervous system development, contactin-2 is involved mainly in neurogenesis. For example, contactin-2 and amyloid precursor protein (APP) are co-expressed in the neurogenic ventricular zone and neural progenitor cells of embryonic day 14 mouse brains, and the contactin-2/APP signaling pathways have been suggested to modulate neurogenesis [4]. After the stage of abundant neurogenesis, contactin-2 becomes expressed by migrating neurons and outgrowing axons. For example, in the rat GABA-expressing interneurons, originating in the medial ganglionic eminence of the ventral telencephalon, migrate along contactin-2-expressing axons of the developing corticofugal system to reach the dorsal telencephalon [5]. Contactin-2 is also strongly expressed on the growth cone of extending axons and secreted into the extracellular matrix, implicating contactin-2 in initiating axon extension and fasciculation and, in addition, guiding axons to their targets by homophilic and heterophilic binding mechanisms [6], [7]. Thereafter, expression of contactin-2 on axons, Schwann cells, and oligodendrocytes appears to become important for myelination and, in particular, for the formation of the juxtaparanodal complex [8], [9]. In the adult rodent brain, expression of contactin-2 is restricted to certain areas exhibiting persistent neural plasticity, e.g. olfactory bulb and hippocampus, and possibly also cerebellar granule cells [10]. These features implicate contactin-2 in plasticity of the adult central nervous system of mammals.

During nervous system regeneration of lower vertebrates, the ventricular zone reinitiates extensive neurogenesis, neural cell migration, axonal regrowth, retargeting and remyelination in a manner very similar to development. Furthermore, upregulation of contactin-2 expression was observed after nervous system injury. For example, re-expression of contactin-2 was found in nasal retinal ganglion cells after zebrafish optic nerve lesion, indicating an active role for contactin-2 in regeneration in a nervous system that is capable of regeneration in the adult [11]. However, functional tests on contactin-2 in nervous system regeneration have not been performed. Our interest in the functional roles of contactin-2 was instigated by the observation that contactin-2 mRNA expression is upregulated following spinal cord injury (SCI) in a microarray analysis of transcripts in the regeneration competent brainstem of adult zebrafish [12].

Adult zebrafish, unlike mammals, exhibit spontaneous spinal cord regeneration after lesion. The availability of a broad repertoire of in vivo experimental techniques, such as morpholino-mediated inhibition of target gene translation, makes the zebrafish an ideal paradigmatic vertebrate model system to test the functions of genes that may be involved in nervous system regeneration [13]. Due to a considerable level of conservation between zebrafish and human genomes, such genes, once identified, could point to possibilities for addressing the multiple issues on functional recovery after SCI and other nervous system disorders in humans.

In the current study, we first verified the results from the microarray analysis and then studied the expression patterns and functions of contactin-2 in spinal cord regeneration. Upregulation of contactin-2 expression was detected after SCI by qPCR, Western blot analysis, in situ hybridization and immunohistology. Inhibition of contactin-2 mRNA translation using a contactin-2 anti-sense morpholino (MO), retarded the recovery of both swimming ability and regrowth, beyond the lesion site, of axons from neurons of the regeneration-competent nucleus of the medial lateral fascicle (NMLF).

## Results

### Contactin-2 mRNA Levels are Upregulated Along the Central Canal after Spinal Cord Injury

**Figure 1:**
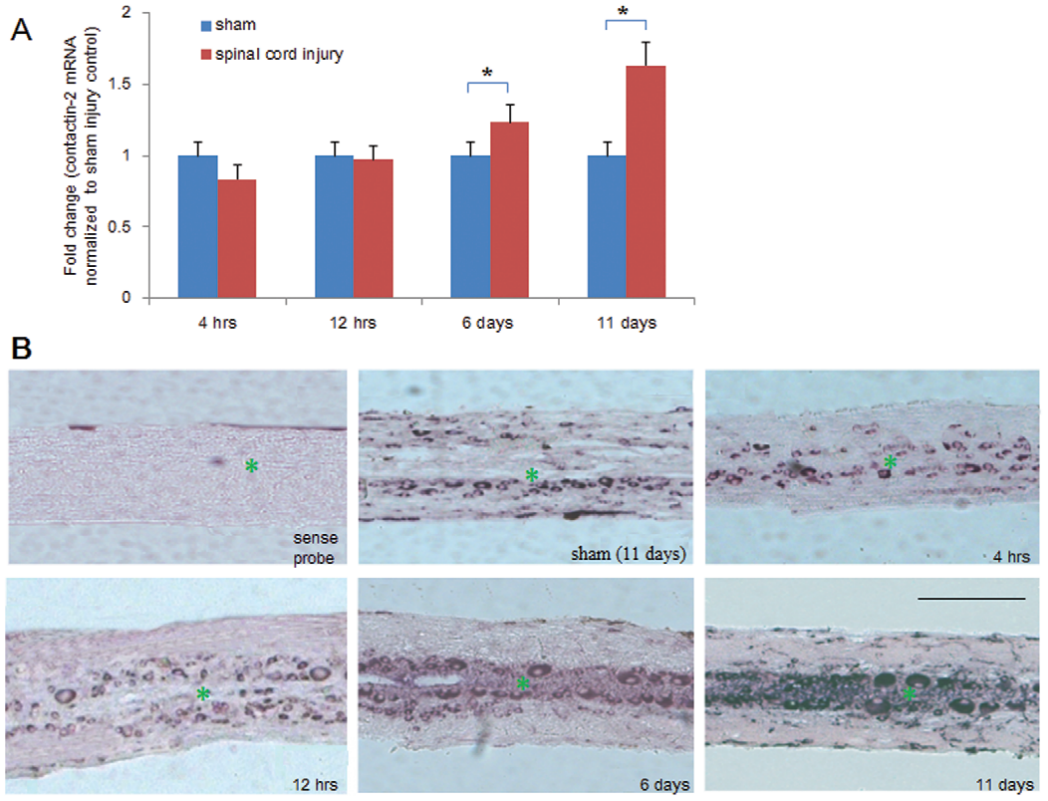
Time course of contactin-2 mRNA expression after spinal cord injury. (A) Quantitative PCR (qPCR) shows expression of contactin-2 mRNA in the spinal cord caudal to the lesion site at four time points after SCI. Significant upregulation was observed at 6 and 11 days after SCI compared to the sham injury group (*p<0.05, one-way ANOVA with Tukey’s post-hoc test; n = 8 fish/group). Values represent means+SEM. (B) In situ hybridization detection of contactin-2 mRNA in longitudinal sections of the spinal cord 3 mm caudal to the lesion site at 4 and 12 hrs, and at 6 and 11 days after SCI (n = 3 fish/group). Numbers of positive cells were increased along the central canal at 6 and 11 days after SCI compared to the sham injury group at 11 days. No signal was detected in sections incubated with a sense probe. * indicates the central canal. Scale bar, 400 µm.

To investigate the involvement of contactin-2 in spinal cord regeneration in adult zebrafish, the expression patterns of contactin-2 in the caudal part of spinal cord after injury were examined by real time qPCR and in situ hybridization at different time points after complete spinal cord lesion. The time points were set at 4 hrs, 12 hrs, 6 days and 11 days after SCI. The mRNA levels of contactin-2 were determined by real time qPCR and normalized to the levels of GAPDH. Since the values of the sham groups at different time points were not significantly different from each other, they were normalized to scale 1. The 4 and 12 hrs represent the acute response phase of the spinal cord to the injury, and the 6 and 11 days represent the more chronic response phase when regeneration occurs. The mRNA levels of contactin-2 remained unchanged at 4 and 12 hrs after SCI, but significantly increased by 1.25-fold at 6 days and 1.75-fold at 11 days after SCI (Figure 1A). The results were confirmed morphologically by in situ hybridization. In the sham injury group (11 days after laminectomy), and in the 4- and 12-hr after injury groups, comparable signals of contactin-2 mRNA were detected in the large cells around the central canal, which are inferred to be motoneurons based on the large size of their cell bodies. At 6 and 11 days after SCI, a significant increase of contactin-2 mRNA was detected along the central canal compared to the sham injury group (Figure 1B). No signal was detected in sections incubated with sense probes under the same conditions as the anti-sense probe, and no significant difference was found between non-injured zebrafish and the sham injury group by qPCR (data not shown).

### Protein Levels of Contactin-2 are Upregulated in the Caudal Part of Spinal Cord after Spinal Cord Injury

**Figure 2:**
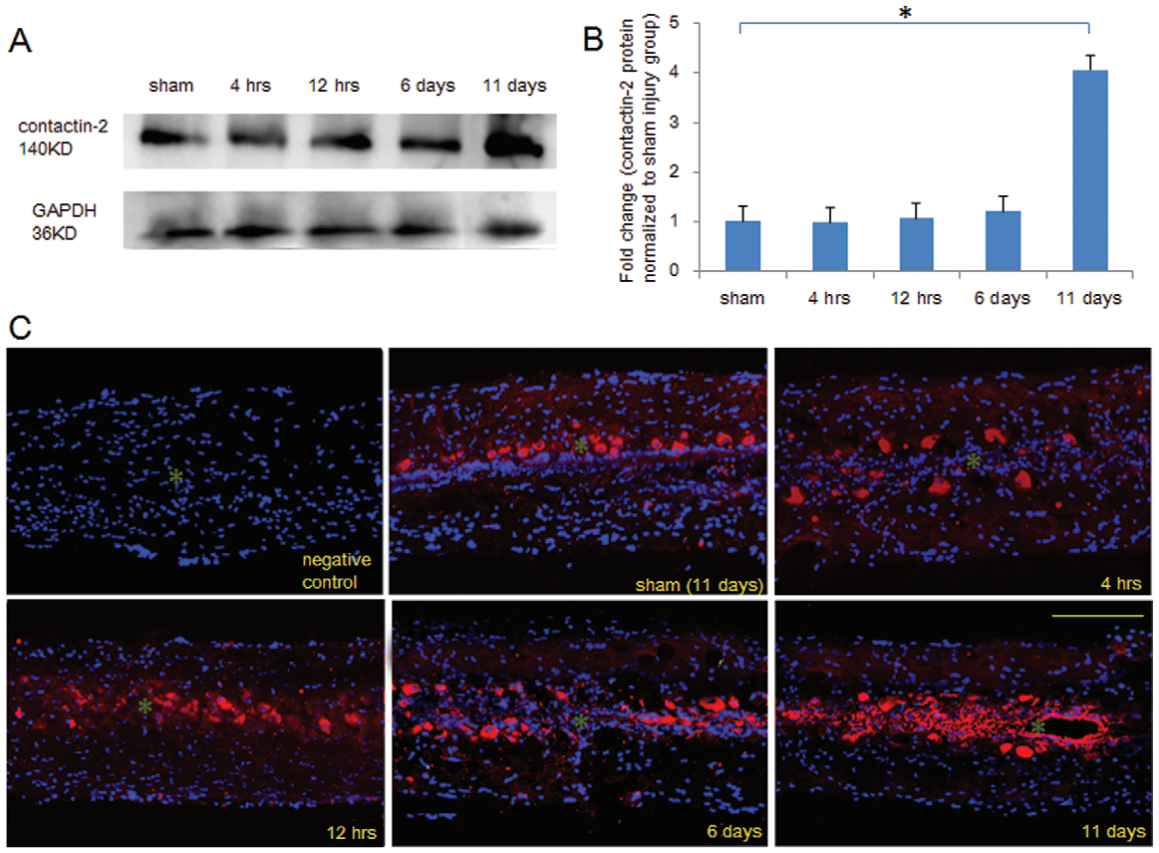
Time course of contactin-2 protein expression after spinal cord injury. (A) Western blot analysis shows contactin-2 protein expression in the spinal cord caudal to the lesion site at four time points after SCI. Significant upregulation was detected at 11 days after SCI compared to the sham injury group at 11 days. (B) The intensity of bands as quantified by ImageJ software and fold change compared with the sham injury group shows an increase in contactin-2 protein level that achieved significance at 11 days after SCI (*p<0.05, one-way ANOVA with Tukey’s post-hoc test; n = 5 fish/group). Values represent means+SEM. (C) Contactin-2 expression in the spinal cord 3 mm caudal to the lesion site was examined by immunofluorescence in longitudinal sections at 4 and 12 hrs, and at 6 and 11 days after SCI. Upregulation of contactin-2 was mainly observed along the central canal at 11 days after SCI. Negative control (without primary antibody), contactin-2 (red), DAPI (blue). * indicates the central canal. Scale bar, 200 µm.

To verify the upregulation of contactin-2 in the caudal part of spinal cord after injury, we studied the protein levels of contactin-2 by Western blot and immunofluorescence analyses. Western blot analysis revealed a characteristic band at around 140 kDa for zebrafish contactin-2 in detergent lysates from spinal cord tissues caudal to the lesion site. Significant upregulation of the 140 kDa band was detected at 11 days after injury (Figure 2A). The intensity of bands was quantified by ImageJ software, and the protein levels of contactin-2 were normalized to GAPDH. Contactin-2 protein levels were increased by approximately 4.1 fold increased at 11 days after SCI, but there was no significant upregulation at 6 days after SCI although mRNA levels were upregulated at the corresponding time point (Figure 2B). These results were confirmed by immunohistology. In the sham injury groups, and the 4- and 12-hr, and the 6-day injury groups, contactin-2 was detected in cells with different cell body sizes, which were inferred to be motoneurons, while additional contactin-2-positive cells emerged along the central canal at 11 days after SCI. No signal was detected in the sections incubated without primary antibody under the same conditions (Figure 2C).

### Contactin-2 Protein is Expressed in Motoneurons and Upregulated Along the Central Canal after Spinal Cord Injury

**Figure 3:**
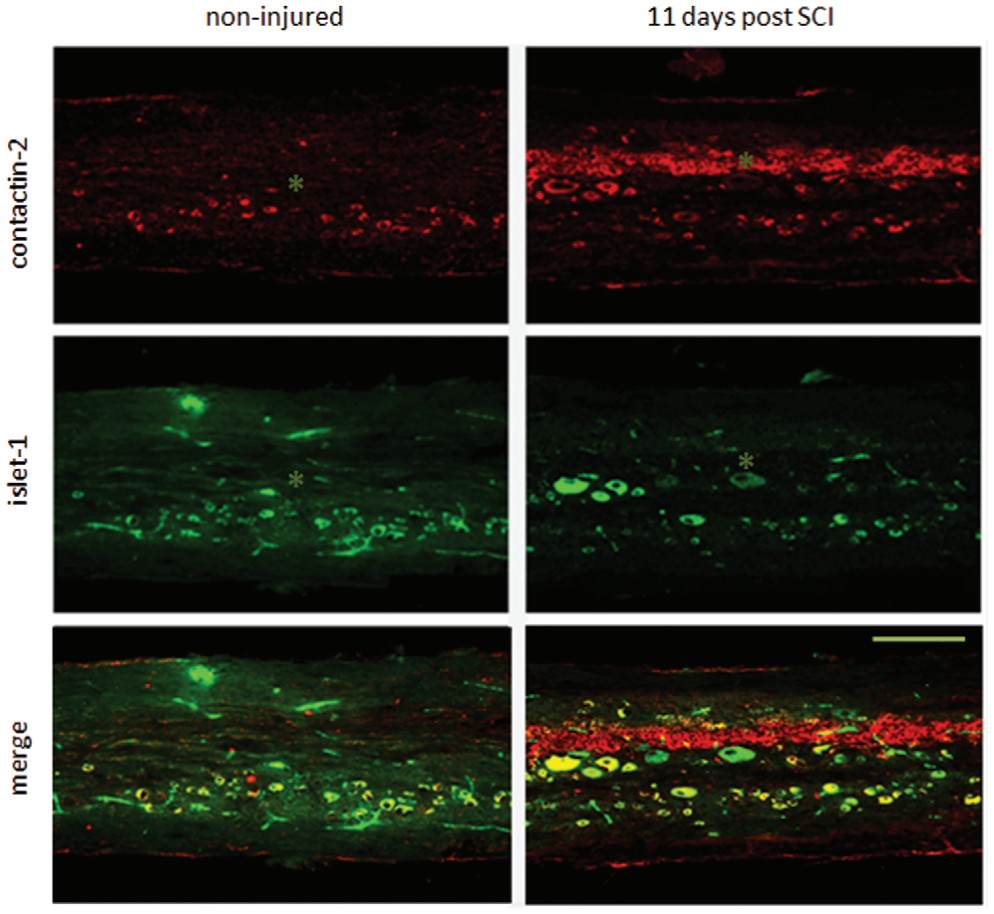
Contactin-2 protein expression is upregulated by motoneurons along the central canal after spinal cord injury. Double immunolabeling of contactin-2 with islet-1 in longitudinal sections (3 mm caudal to the lesion site) shows that in the spinal cord of non-injured zebrafish, contactin-2 is prominently expressed by motoneurons and other cell types. At 11 days after SCI, contactin-2 expression is increased compared to non-injured fish along the central canal. * indicates the central canal. Scale bar, 200 µm.

To investigate whether motoneurons express contactin-2, double immunostaining was performed for contactin-2 and islet-1 (a marker for motoneurons) in non-injured and injured zebrafish. In the spinal cords of non-injured adult zebrafish, most of the contactin-2 signal co-localized with islet-1 (Figure 3). In the 11-day SCI groupwe observed, in addition to the normally high level of contactin-2 expression in motoneurons, a significant upregulation of contactin-2 expression along the central canal (Figure 3).

### Knock Down of Contactin-2 Expression after Spinal Cord Injury Retards Locomotor Recovery after Spinal Cord Injury

**Figure 4:**
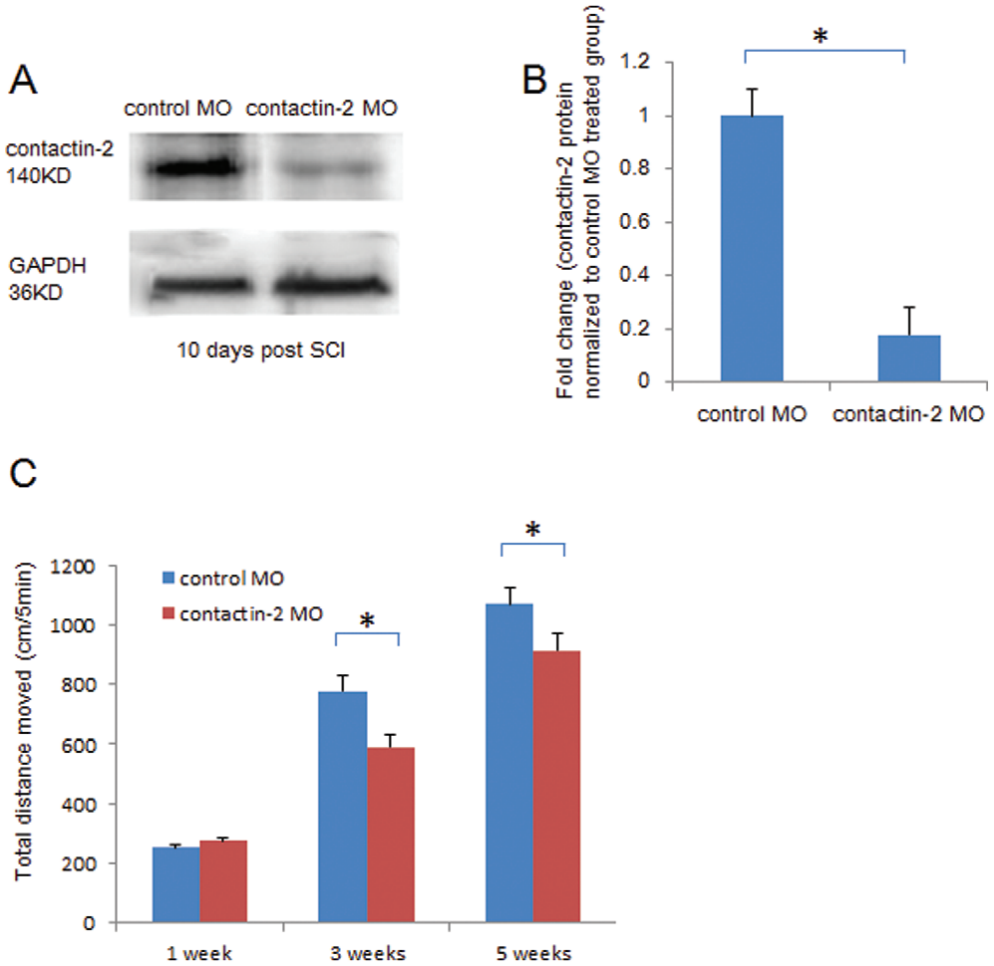
Knock down of contactin-2 expression after spinal cord injury retards locomotor recovery after spinal cord injury. (A) Contactin-2 protein expression is reduced at 10 days after SCI by application of contactin-2 antisense morpholino (MO) as detected by Western blot analysis. (B) Intensity of the bands as quantified by ImageJ software, and fold change compared with control MO treated group shows a decrease in contactin-2 protein level at 10 days after application of contactin-2 MO (*p<0.05, Student’s t-test; n = 5 fish/group). Values represent means+SEM. (C) Total distance moved in 5 min of undisturbed zebrafish treated with contactin-2 MO or control MO (n = 8 fish/group) after SCI. At 3 and 5 weeks after SCI, the total distance moved was reduced by treatment with contactin-2 MO when compared to fish treated with control MO (*p<0.05, one-way ANOVA with post-hoc Student-Newman-Keuls test; n = 8 fish/group). Values represent means+SEM.

To further elucidate the importance of contactin-2 upregulation in spinal cord regeneration, we knocked down contactin-2 expression with an anti-sense MO, and tracked the recovery of swimming ability. Contactin-2 anti-sense MO significantly reduced contactin-2 protein levels by approximately 82% compared with control MO 10 days after MO treatment being set to 100% (Figures 4A and B). Even 3 weeks after MO treatment, contactin-2 protein levels continued to remain decreased to a level of 49% (data not shown). It is noteworthy in this context that MO inhibition has been observed to last for more than 6 weeks [14].

To analyze the effect of contactin-2 knock down on functional recovery after SCI, we quantified locomotor recovery as described [15]. After SCI, the total distance moved by undisturbed fish, during a period of 5 min, was evaluated weekly by automatic video recording. At one week after SCI, the swimming ability of fish from both the control MO and contactin-2 MO treated groups was strongly inhibited, with the total distance moved being strongly reduced to about 10% of that seen in non-injured fish (data not shown). Starting from 3 weeks after SCI, the total swimming distance from the control MO treated group was considerably longer than that from the contactin-2 MO treated group. Significant differences between control and anti-sense MO were found at 3 and 5 weeks after SCI (Figure 4C).

### Reduction of Contactin-2 Protein Expression after Spinal Cord Injury Impairs Axon Regrowth Beyond the Injury Site

**Figure 5:**
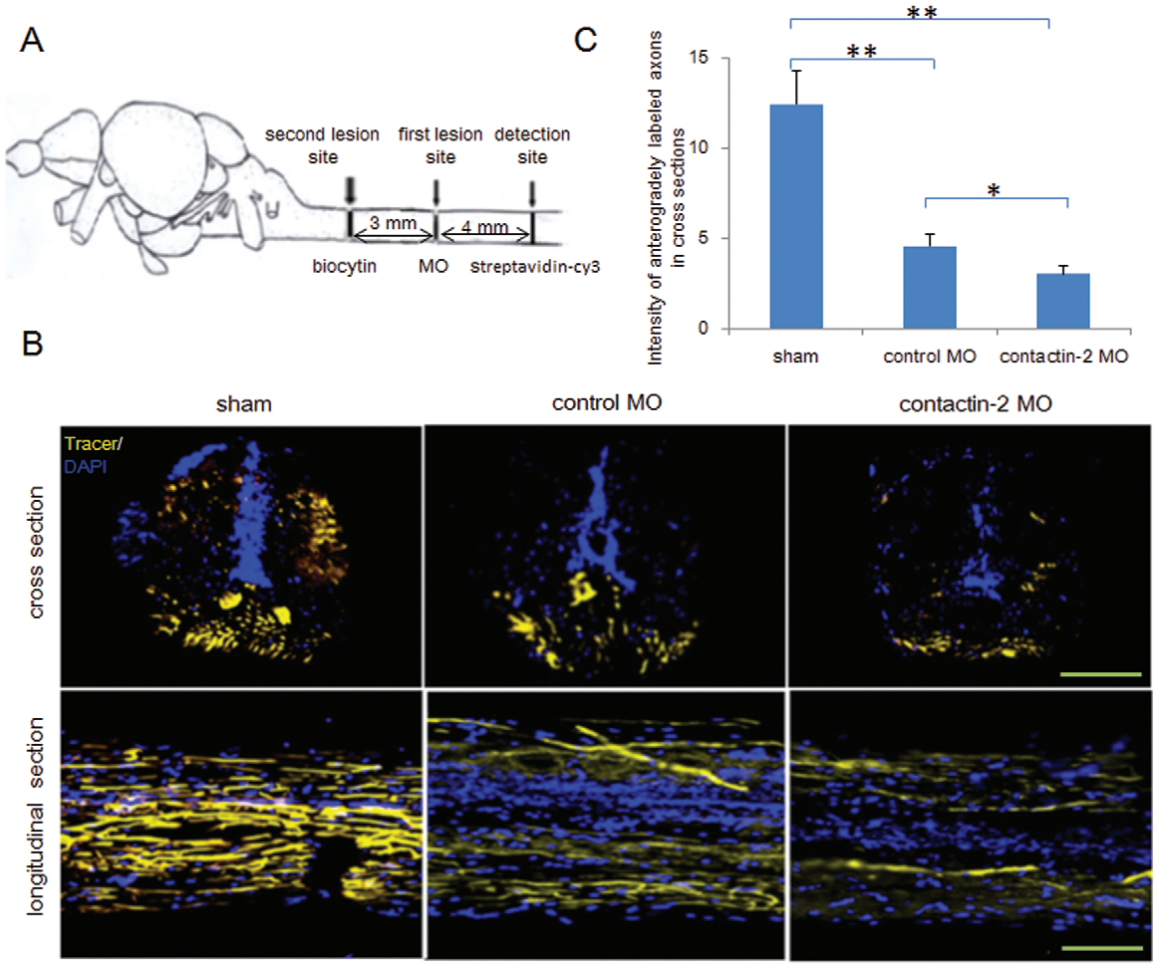
Reduction of contactin-2 protein expression after spinal cord injury impairs regrowth of axons. (A) Schematic illustration for anterograde tracing. Biocytin was applied at the site symbolized by the second lesion site at 6 weeks after the first lesion. After 24 hrs, biocytin was detected at the site 4 mm distal to the first lesion site by Streptavidin-Cy3. (B) Cross and longitudinal sections show axons regrown beyond the first lesion site. (C) Relative fluorescence intensity for cross sections was quantified by ImageJ software. A 34% decrease in intensity was observed in the contactin-2 anti-sense MO treated group compared to the control MO treated group (*p<0.05, **p<0.01, t-test; n = 3 fish/group). The sham injury group shows similar intensities as the non-injury group. Scale bars, 200 µm.

To elucidate the functions of contactin-2 in spinal cord regeneration, we studied axonal regrowth beyond the lesion site by anterograde tracing. Biocytin was applied at the site symbolized by the second lesion site at 6 weeks after the first lesion (Figure 5). After 24 hrs, biocytin was detected by Streptavidin-Cy3 at a site 4 mm distal to the first lesion site (Figure 5A). Axon regrowth beyond the first lesion site was estimated in cross and longitudinal sections (Figure 5B). The intensity of signal in cross sections was quantified by imageJ software and compared with the sham injury group (Figure 5C). Compared to the sham lesion group (set to 100%), the intensity of the signal in the control MO treated group was found to be 37%, while in contactin-2 MO treated group the value was 24%. This indicated that only approximately 37% axons from the control MO treated group and approximately 24% axons from the contactin-2 MO treated group had regrown beyond the lesion site at 6 weeks after SCI. In conclusion, knock down of contactin-2 impairs axonal regrowth after SCI.

### Reduction of Contactin-2 Protein Expression after Spinal Cord Injury Impairs NMLF Neurons to Regrow their Axons Beyond the First Lesion Site

**Figure 6:**
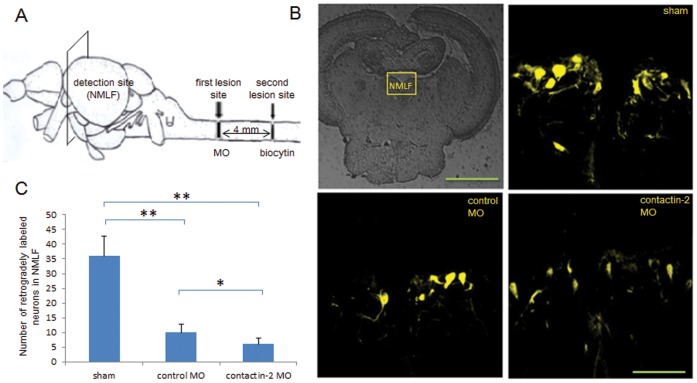
Reduction of contactin-2 protein expression after spinal cord injury impairs axon regrowth of NMLF neurons. (A) Schematic illustration for retrograde tracing. Biocytin was applied at the site symbolized by the second lesion site at 6 weeks after the first lesion, and was detected in the NMLF after 24 hrs using Streptavidin-Cy3. (B) Retrogradely labeled neurons in the NMLF. (C) Numbers of retrogradely labeled neurons in the NMLF. Reduction of contactin-2 protein expression by application of contactin-2 anti-sense MO reduces the numbers of NMLF neurons with axons regrown beyond the first lesion site when compared to the control MO treated group (*p<0.05, t-test; n = 3 fish/group). The sham injury group shows similar numbers of retrogradely labeled neurons as the non-injury group. Scale bars, 200 µm and 50 µm.

By retrograde tracing, we specifically labeled the cell bodies of NMLF neurons that regrow their axons beyond the lesion site at 6 weeks after SCI (A schematic illustration for retrograde tracing is shown in Figure 6A). All labeled neurons in the NMLF were thus determined in serial sections (Figure 6C). The number of labeled neurons in control MO treated (10±2.9/fish) and contactin-2 MO treated (6±2.1/fish) groups was significantly less compared with the sham injury group (36±6.8/fish). Thus, knock down of contactin-2 impairs NMLF neurons to regrow their axons beyond the injury site at the 6 weeks after SCI.

## Discussion

### Expression Patterns of Contactin-2 in the Adult Central Nervous System

Extensive studies have been performed on the expression patterns and functions of contactin-2 in nervous system development. However, fewer have focused on the adult nervous system. In the adult mouse brain, contactin-2 was restricted in expression to certain regions that experience persistent neural plasticity, such as the olfactory bulb, hippocampus, and cerebellar granule cells [10]. In the adult zebrafish and goldfish, contactin-2 was detected in the newly generated nasal retinal ganglion cells, which are continuously generated around the margin of the retina [11], suggesting possible functions in plasticity. Our study revealed that in the spinal cord of adult zebrafish, contactin-2 mRNA and protein were expressed in motoneurons of the non-injured zebrafish which is in accordance with the expression of contactin-2 in motoneurons in the spinal cord of the adult rat, where the molecule’s function remains to be determined [16]. Since contactin-2 is not only expressed in the spinal cord after SCI, but also before SCI, we consider it unlikely that contactin-2 derived from motoneurons is a major contributor to the remodeling of synaptic contacts of regrowing descending and ascending axons with motoneurons, although upregulation of its expression after injury could signify that it serves a function in motoneuronal neurite outgrowth after injury that would contribute to regeneration. It is also conceivable that contactin-2 contributes to the maintenance of synaptic contacts.

The cytoplasmic expression of contactin-2 in motoneurons is considered to be the source of contactin-2 that is secreted into either the extracellular matrix or cerebrospinal fluid [3]. Secreted contactin-2 in the extracellular matrix is a potent substratum for neurite outgrowth [17]. The functions of contactin-2 in the cerebrospinal fluid, however, remain unknown.

### Regulation of Contactin-2 Expression in Response to Nervous System Injury

In the rat, contactin-2 expression is no more detectable in retinal ganglion cells within 2 days after optic nerve lesion and is not re-expressed neither in the cell bodies nor regrowing axons of retinal ganglion cells [18]. In a rat sciatic nerve lesion model, contactin-2 expression is down-regulated in dorsal root ganglion neurons but upregulated in reactive Schwann cells with time after injury [16]. In contrast, in adult zebrafish, re-expression of contactin-2 is observed in axotomized retinal ganglion cells after optic nerve lesion [11], and microarray analysis revealed upregulation of contactin-2 mRNA in NMLF neurons at 11 days after SCI [12]. In the present study, we found that contactin-2 is highly expressed in motoneurons before and after injury. The higher levels of contactin-2 expression in neurons of adult zebrafish rather than the adult rodent animals before and after central nervous system injury may be responsible for the more pronounced regeneration seen in zebrafish, since both the secreted contactin-2 and membrane attached contactin-2 can promote neurite outgrowth [17], [19]. By anterograde and retrograde axon tracing, we found that inhibition of contactin-2 expression with a contactin-2 anti-sense MO at the time of SCI robustly inhibited axon regrowth beyond the lesion site, indicating a positive role of contactin-2 in promoting axonal regrowth. A possible mechanism is that knock down of contactin-2 expression inhibits the interaction of contactin-2 with the neural cell adhesion molecule L1 (L1.1 in zebrafish), which was shown to be involved in axonal regeneration of lesioned adult zebrafish [14]. It was also shown that the interaction of contactin-2 with L1 promotes neurite outgrowth in vitro [20], [21]. Since contactin-2 acts by homophilic and heterophilic mechanisms that are conducive to neurite outgrowth, secreted contactin-2 may use these mechanisms for recovery under condition of traumatic injury, particularly in view of the possibility that proteases are activated under conditions of trauma.

Prominent upregulation of contactin-2 expression was found along the central canal starting from 6 days following SCI. Upregulation of contactin-2 expression along the central canal is most likely involved in the neurogenesis of ependymo-radial glial cells after SCI. These cells begin to migrate away from the lesion site at 3 days after injury, and are still detected at the injury site up to 7 days after injury [22]. At one to two weeks after injury, extensive neurogenesis has been reported in the ventricular zone [23], which coincides with the time of upregulation of contactin-2 protein expression along the central canal. A recent study on brain stab lesions in adult zebrafish shows that, during the recovery phase, radial-glia type progenitors that had migrated to the lesion site and proliferated after the lesion contribute to the population of newly generated neurons which could survive for more than 3 months [24]. Expression of contactin-2 by subsets of developing embryonic neurons has been observed [25]–[27] and it will be interesting to analyze the expression of contactin-2 in subsets of spinal cord neurons, once appropriate tools for the detection of these subsets become available.

The contribution of contactin-2 to axon fasciculation in development has been extensively studied. Since in addition to neurons, contactin-2 is also expressed by central nervous system glia, it is possible that down-regulation of contactin-2 expression in these cells also contributes to the reduced regenerative capacity in contactin-2 MO treated fish. By using transgenic mice that exclusively express TAG-1 in proteolipid-expressing mature oligodendrocytes it could be shown that TAG-1 restores the axonal and myelin deficits seen in constitutively Tag-1 (contactin-2) deficient mice [28]. Thus, reduced expression in oligodendrocytes may impair their ability to remyelinate regrown axons, thereby maintaining trophic support for axons. Reduced expression in astrocytes may impair their capacity to interact with neuronal structures for promotion of regrowth of severed axons and remodeling of dendrites via the astrocytic substrate.

The common features between nervous system development and regeneration in adult zebrafish, as well as the upregulation of contactin-2 expression after SCI indicate an active role for contactin-2 in regeneration after central nervous system injury. In this study, we showed that contactin-2 is involved in axonal regrowth during spinal cord regeneration. Whether the upregulation of contactin-2 expression along the central canal would suggest more complex and beneficial roles in regeneration remains to be seen.

### Conclusions

In this study, upregulation of contactin-2 expression in the spinal cord caudal to the lesion site in response to spinal cord injury was confirmed by qPCR and Western blot analysis, and upregulation of expression was observed along the central canal by in situ hybridization and immunohistology. Inhibition of contactin-2 expression by application of contactin-2 anti-sense morpholino retards the recovery of swimming ability and axonal regrowth of NMLF neurons beyond the lesion site, indicating that contactin-2 is beneficial for functional recovery, at least in part through promoting axonal regrowth.

## Materials and Methods

### Animals

Adult zebrafish (*Danio rerio*, 6 months old) were purchased from Huiyuan Aquatic Animals Company (Shantou, Guangdong, China). Fish were kept on a 14 hr light and 10 hr dark cycle at 28°C. All animal experiments were approved by the Animal Ethics Committee of Shantou University Medical College.

### Spinal Cord Injury

Spinal cord transection was performed as described [14], [15], [29]. Briefly, fish were anaesthetized by immersion in 0.033% aminobenzoic acid ethylmethylester (Sigma, St. Louis, MO, USA) in phosphate buffered saline (PBS, pH 7.4) for 5 min. A longitudinal incision was made at the side of the fish to expose the vertebral column. The spinal cord was cut between two vertebrae, about 4 mm caudal to the brainstem/spinal cord transitional junction. Wounds were sealed with Histoacryl (B. Braun, Melsungen, Germany), and the injured fish were kept individually. A sham control was set by making the incision, but without spinal cord transection. All surgical procedures were performed on ice under a microscope.

### Real Time Quantitative RT-PCR (qPCR)

To study the expression of contactin-2 mRNA in the spinal cord caudal to the lesion site, total RNA was extracted, from the 4 mm piece of spinal cord caudal to the lesion site, at different time points after SCI. First-strand cDNA was generated using random primers and ReverTraAce^R^ qPCR RT Kit (TOYOBO, Osaka, Japan). Quantitative real time polymerase chain reaction (qPCR) was performed with SYBR^R^ Green Realtime PCR Master Mix (TOYOBO, Osaka, Japan) as described [30]. Primers for qPCR were designed using Primer Premier 5.0 software (Premier Biosoft International, Palo Alto, CA, USA). All assays were performed in duplicate samples, and assay products were validated using melting curves to confirm the presence of single PCR products. GAPDH served as the internal control. The following primer sequences were used: zebrafish contactin-2 forward: TTCGGCTACCTGCGTGAT, reverse: TGATAAACCAGCGGTAGGA. GAPDH forward: GTGTAGGCGTGGACTGTGGT, reverse: TGGGAGTCAACCAGGACAAATA [31].

### In Situ Hybridization

*In situ* hybridization probes (sense and anti-sense) for contactin-2 mRNA (NM-131446.1) were transcribed *in vitro*. Purified PCR fragments were cloned into the pGM-T vector (Tiangen, Beijing, China), and the sequences were verified by sequencing. Digoxigenin (DIG)-labeled sense and anti-sense RNA probes were generated using the Megascript system (Ambion, Austin, TX, USA) according to the manufacturer’s instructions. Non-radioactive detection of mRNA in sections of the adult zebrafish central nervous system was performed as described [32]. Spinal cords were fixed for 24 hrs in 4% paraformaldehyde in PBS at 4°C, followed by incubation in 15% sucrose in PBS overnight at 4°C. Then, 16 µm-thick sections of spinal cords were cut from fresh-frozen tissue on a cryostat, pre-hybridized for 2 hrs at 55°C, and hybridized with the DIG-labeled probes at 55°C overnight. After extensive washing (at 50°C), alkaline phosphatase-coupled anti-DIG fragment antibodies (Fabs, Roche, Indianapolis, IN, USA) were applied at room temperature for one hr. Antibody binding was detected using an alkaline phosphatase reaction with nitro-blue-tetrazolium and 5-bromo-4-chloro-3-indolyl phosphate (NBT/BCIP, Roche Indianapolis, IN, USA) as substrates. For negative control, sense probes were developed in parallel under the same conditions as the anti-sense probes. Sections from sham control and injured fish were analyzed on the same slides. Sections were viewed and photographed using an epifluorescence microscope (Axio Imager Z1, Zeiss, Oberkochen, Germany).

### Immunohistochemistry

All spinal cord tissues were processed for immunofluorescence after fixation in 4% formaldehyde in PBS at 4°C overnight. Serial sections (16 µm thick) from spinal cord (longitudinal, 0–4 mm caudal to lesion site) were used. Sections were prepared as described above and processed for immunostaining [15]. The primary antibodies were rabbit anti-TAG-1 (1∶800, [11]), mouse anti-islet-1 (1∶100, Developmental Studies Hybridoma Bank, University of Iowa, Iowa City, IA, USA). Secondary antibodies were Alexa Fluor CY3 goat anti-rabbit IgG (1∶800, Jackson ImmunoResearch) and FITC goat anti-mouse IgG (1∶800, Jackson ImmunoResearch). Fluorescent images were taken using an epifluorescence microscope (Axio Imager Z1, Zeiss, Oberkochen, Germany).

### Western Blot Analysis

Total protein was extracted by homogenizing the spinal cord tissue in Radioimmunoprecipitation Assay (RIPA) lysis buffer (Soliba, Beijing, China). After centrifugation of the homogenate at 14000×g and 4°C, the protein concentration in the supernatant was determined with a BCA kit (Pierce, Rockford, USA). The supernatant was denatured by boiling for 5 min in SDS sample buffer. Fifty micrograms of total protein was then subjected to SDS-PAGE on 8% gels. Proteins were transferred to a polyvinylidene difluoride membrane and probed with the following antibodies: rabbit anti-TAG-1 (1∶2000, [11] ) and mouse anti-GAPDH (1∶1000, Beyotime, Haimen, China). Goat anti-rabbit IgG and goat anti-mouse IgG (1∶1000, Jackson ImmunoResearch) conjugated to horseradish peroxidase were used as secondary antibodies. The grey value of each band was measured and normalized to that of GAPDH band by ImageJ software (NIH).

### Morpholino Treatment

MOs are synthetic molecules that are the product of a redesign of natural nucleic acid structures. MOs targeting the start codon region can interfere with the progression of the ribosomal initiation complex from the 5′ cap to the start codon and prevent translation of the coding region of the targeted transcript, thus decreasing the protein expression level [33]. Contactin-2 anti-sense MO (5′-GACACAACAGAATCCTCATCCTCAT-3′, vivo-porter-coupled) and standard control MO (5′-CCTCTTACCTCAGTTACAATTTATA-3′, vivo-porter-coupled) (Gene Tools, Philomath, OR, USA) were dissolved in Danieau solution [34] and soaked onto small pieces of Gelfoam (Upjohn, Kalamazoo, MI, USA). These pieces of Gelfoam were divided into smaller pieces to yield 800 ng of MOs, approximately 0.22 µl per piece, and allowed to dry. One piece was applied to the transection site immediately after spinal cord transection. Each animal was allowed to survive the surgery for 6 weeks.

### Swim Tracking

Swimming capacities of injured fish were assessed each week after MO application for 6 weeks in two trials of 5 min each (trial interval, 4 hrs). In each trial, a zebrafish was placed in a brightly illuminated (100 lux) tank (42×30×30 cm) filled with aquarium water (5 cm deep) at 25°C. A video camera recorded the trials from above the tank. Swim paths were tracked with Ethovision software (Noldus, Wageningen, Netherlands). Mean lengths of the swim paths (total distance moved) of the two trials were used for graphical presentation and statistical analysis. The experimenter was blinded to the treatment of the animals.

### Retrograde Tracing

Retrograde axonal tracing was performed by application of tracer biocytin (Sigma, St. Louis, MO, USA) 4 mm caudal to the spinal lesion site 6 weeks after transection (see Fig. 6 for illustration) [14], [29], [35]. In brief, Gelfoam pieces soaked in a saturated solution of biocytin were left at the lesion site for the tracer to be retrogradely transported. Twenty four hrs after application, brains were dissected and fixed in 4% formaldehyde in PBS overnight and embedded in 15% sucrose at 4°C. Serial coronal brain sections (30 µm thick) were obtained using a cryostat. Biocytin labeling in the sections was detected with Streptavidin-Cy3 (1∶200, Bioss). Labeled neurons in the whole nucleus of medial longitudinal fascicle (NMLF) were counted, in serial brainstem sections, using an epifluorescence microscope (Axio Imager Z1, Zeiss, Oberkochen, Germany) by an investigator blind to treatment. Three animals were in each group (treated with control MO or contactin-2 MO).

### Anterograde Tracing

To study pre-synaptic endings from regenerating axons past the lesion site, biocytin was applied at the brainstem/spinal cord junction through a second surgery 6 weeks after SCI (see Figure 5 for illustration) [14], . After 24 hrs, spinal cords were removed, and tissue, 0–4 mm in length caudal to the original spinal lesion site, was fixed in 4% formaldehyde in PBS at 4°C overnight, embedded in 15% sucrose, and sectioned (20 µm-thick sections) coronally on a cryostat. Biocytin labeling was detected with Streptavidin-Cy3. Fluorescence intensity is presented as the mean intensity from 12 randomly selected sections of spinal cords 0–4 mm caudal to the lesion site. Three animals were in each group (treated with control MO or contactin-2 MO).

### Statistical Analysis

Fluorescence intensity was analyzed by ImageJ software (NIH). Using SPSS 13 software, the Student′s *t*-test was used for comparing two treatment groups, and one-way ANOVA of variance was used for multiple group comparisons. Data are presented as means+SEM. *p*<0.05 is set as indicating significance (*, for *p*<0.05; **, for *p*<0.01). All experiments were performed 3 independent times.
